# Supplementary material for: Single-layer spatial analog meta-processor for imaging processing
Source: Nat Commun. 2022 Apr 21;13:2188. doi: 10.1038/s41467-022-29732-4 (PMC9023575; doi:10.1038/s41467-022-29732-4)
Supplement: Supplementary file 1 — Supplementary Information [file 41467_2022_29732_MOESM1_ESM.pdf]

Supplementary Information

Single-layer spatial analog meta-processor for imaging processing

Zhuochao Wang,[1†] Guangwei Hu[2†], Xinwei Wang[1†], Xumin Ding[1*], Kuang Zhang[3*], Haoyu Li[1*], Shah Nawaz Burokur[4*], Qun Wu[3], Jian Liu[1], Jiubin Tan[1] and Cheng-Wei Qiu[2*]

[1]Advanced Microscopy and Instrumentation Research Center, School of Instrumentation Science and Engineering, Harbin Institute of Technology, Harbin 150080, China

[2] Department of Electrical and Computer Engineering, National University of Singapore, Singapore 117583

[3]Department of Microwave Engineering, Harbin Institute of Technology, Harbin 150001, China

[4]LEME, UPL, Univ Paris Nanterre, F92410 Ville d'Avray, France

† Zhuochao Wang, Guangwei Hu and Xinwei Wang contributed equally to this work.

Correspondence and requests for materials should be addressed to X.D. (email: xuminding@hit.edu.cn) or to K.Z. (email: zhangkuang@hit.edu.cn) or to H.L. (email: lihaoyu@hit.edu.cn) or to S.B. (email: sburokur@parisnanterre.fr) or to C.W.Q. (email: chengwei.qiu@nus.edu.sg).

Supplementary Note 1 The benchmark table of metastructure-based analog processors

Supplementary Note 2 Derivation of the aperture function on Huygens' metasurface processor

Supplementary Note 3 Design of Huygens' meta-atom structure

Supplementary Note 4 The wavefront profiles on Huygens' metasurface for edge detection

Supplementary Note 5 The resolution of the first-order derivative and cross-correlation operation

Supplementary Note 6 Influence of phase and amplitude discretization on the performance of 2D-edge detection

Supplementary Note 7 The wavefront profiles on Huygens' metasurfaces for cross-correlation on one-dimensional sequence

Supplementary Note 8 Application of Huygens' metasurface processor at optical frequencies

Supplementary Note 9 Photograph of the fabricated Huygens' metasurfaces

Supplementary Note 1 The benchmark table of metastructure-based analog processors

To address the novelty and significance, the benchmarks of our work in contrast to other categories of up-to-date metastructure-based analog processors are listed in terms of working mechanism, device design, device dimension, computing throughput, function diversity, as listed in Supplementary Table 1. Particularly, according to the digital throughput measured in bits per second (bit/s) , the throughput of analog processor here is evaluated by the processing range of input data per unit of time.

Firstly, both the GF kernel and our proposed processor revel a distinct advantage in device integration for the single-layer spectrum modulation within sub-wavelength scale. Secondly, compared with the increase of waveguide number with more complicated inverse design of metastructure and the device optimization of GF kernel for wider modulation range of incident angle, the computing throughput can boost enormously by simply increasing the metaatom number of 4*f* Fourier system and our proposed metasurface. Finally, compared with other three methods, the angle-dependent response of GF kernel is suitable for limited Fourier-domain operations (such as derivative and integral). For instance, the differentiation is transformed into the easier multiplication in angular-scattering spectrum, as $E_H\left(k_x\right) \propto jk_x$, which can be implemented by the mechanism of Fano resonance or surface plasmon polariton (SPP). However, for the mathematical function with drastic fluctuations, such as the cross-correlation in our work as $E_{\mathrm{H}}\left(k_x,k_y\right) \propto \left[L_x \frac{k}{f_2} \mathrm{sinc}\left(L_x k_x\right)\right] \times \left[L_y \frac{k}{f_2} \mathrm{sinc}\left(L_y k_y\right)\right]$, the difficulties in angle-dependent metaatom design will be greatly increased. Overall, our proposed meta-processor renders a high level of aggregation with respect to the integration, throughput, function diversity.

Supplementary Table 1. Benchmarks of the recently proposed metastructure-based analog processors. The cases highlighted in pale green color are the best implementable computing performances with specific methods compared with others.

	**The inverse-designed computational metastructure**	**4*f* Fourier system**	**Green's Function (GF) kernel**	**Our work**
**Working mechanism**	Spatial-domain calculation	Spatial-frequency-domain calculation	Angular-scattering spectrum calculation	Spatial-frequency-domain calculation
**Device design**	Inverse-designed metamaterial structure	Two graded refractive index (GRIN) lenses and metasurface	Angle-dependent device	Single-layer metasurface
**Device dimension/ thickness**	Several wavelength scale	Three layers	**Single layer**	**Single layer**
**Function diversity**	**Versatile spatial-domain function types**	**Versatile Fourier-domain function types**	Basic Fourier-domain operations	**Versatile Fourier-domain function types**

Supplementary Note 2 Derivation of the aperture function on Huygens' metasurface processor

Under paraxial approximation and in Fresnel regime, the wave in output plane (dubbed as E_1), regarding the impinging image (E_0) through the Huygens' metasurface (E_{meta}), can be expressed as

$$E_1(x_1,y_1)=-\frac{k^2}{4\pi^2 f_1 f_2}\exp\left[ik(f_1+f_2)\right]\iint_{\Sigma_{meta}} dx'dy'\left\langle \iint_{\Sigma_0} dx_0 dy_0 E_0(x_0,y_0)\exp\left\{\frac{ik}{2f_1}\left[(x'-x_0)^2+(y'-y_0)^2\right]\right\}\right\rangle$$
$$E_{meta}(x',y')\exp\left\{\frac{ik}{2f_2}\left[(x_1-x')^2+(y_1-y')^2\right]\right\}$$
$$=-\frac{k^2}{4\pi^2 f_1 f_2}\exp\left[ik(f_1+f_2)\right]\exp\left(\frac{ik}{2f_2}x_1^2+\frac{ik}{2f_2}y_1^2\right)\iint_{\Sigma_0} dx_0 dy_0 E_0(x_0,y_0)\exp\left(\frac{ik}{2f_2}x_0^2+\frac{ik}{2f_2}x_0^2\right)$$
$$\iint_{\Sigma_{meta}} dx'dy' E_{meta}(x',y')\exp\left[ik\left(\frac{x_0}{f_1}+\frac{x_1}{f_2}\right)x'+ik\left(\frac{y_0}{f_1}+\frac{y_1}{f_2}\right)y'\right]\exp\left\{\frac{ik}{2}\left(\frac{1}{f_1}+\frac{1}{f_2}\right)\left[x'^2+y'^2\right]\right\} \quad (1)$$

From Equation S1, to construct the Fourier transform function in form of $\exp\left[ikx'+iky'\right]$, the quadratic term $\exp\left\{\frac{ik}{2}\left(\frac{1}{f_1}+\frac{1}{f_2}\right)\left[x'^2+y'^2\right]\right\}$ should be eliminated by introducing the concave-lens phase factor $\exp\left[-\frac{ik}{2f}\left(x'^2+y'^2\right)\right]$ on the Huygens' metasurface aperture, where $\frac{1}{f}=\frac{1}{f_1}+\frac{1}{f_2}$. Hence, the the aperture function on the Huygens' metasurface is defined as

$$E_{meta}(x',y')=\exp\left[-\frac{ik}{2f}\left(x'^2+y'^2\right)\right]E_H(x',y') \quad (2)$$

where $\frac{1}{f}=\frac{1}{f_1}+\frac{1}{f_2}$. Then, Equation (1) can be derived as:

$$E_1(x_1,y_1)=-\frac{k^2}{4\pi^2 f_1 f_2}\exp\left[ik(f_1+f_2)\right]\exp\left(\frac{ik}{2f_2}x_1^2+\frac{ik}{2f_2}y_1^2\right)\iint_{\Sigma_0} dx_0 dy_0 E_0(x_0,y_0)\exp\left(\frac{ik}{2f_2}x_0^2+\frac{ik}{2f_2}y_0^2\right)$$
$$\iint_{\Sigma_{meta}} dx'dy' E_H(x',y')\exp\left[ik\left(\frac{x_0}{f_1}+\frac{x_1}{f_2}\right)x'+ik\left(\frac{y_0}{f_1}+\frac{y_1}{f_2}\right)y'\right]$$
$$=-\frac{k^2}{2\pi f_1 f_2}\left\{\exp\left[ik(f_1+f_2)\right]\exp\left(\frac{ik}{2f_2}x_1^2+\frac{ik}{2f_2}y_1^2\right)\right\}\iint_{\Sigma_0} dx_0 dy_0 E_0(x_0,y_0)\exp\left(\frac{ik}{2f_2}x_0^2+\frac{ik}{2f_2}y_0^2\right)$$
$$\mathcal{F}\left\{E_H(x',y')\right\}\left[\frac{k}{f_1}(x_0-\tilde{x}_1),\frac{k}{f_1}(y_0-\tilde{y}_1)\right]$$
$$=-\frac{k^2}{2\pi f_1 f_2}\{...\}\left\{E_0(\tilde{x}_1,\tilde{y}_1)\exp\left(\frac{ik}{2f_2}\tilde{x}_1^2+\frac{ik}{2f_2}\tilde{y}_1^2\right)\right\}\odot\mathcal{F}\left\{E_H(x',y')\right\}\left[-\frac{k}{f_1}\tilde{x}_1,-\frac{k}{f_1}\tilde{y}_1\right] \quad (3)$$

where $\tilde{x}_1=-\frac{f_1}{f_2}x_1$, $\tilde{y}_1=-\frac{f_1}{f_2}y_1$ and $\odot$ represents two-dimensional convolution operation. Via applying the convolution theorem,, described as $\mathcal{F}\left\{f(x)\odot g(x)\right\}=\mathcal{F}\left\{f(x)\right\}[k_x]\times\mathcal{F}\left\{g(x)\right\}[k_x]$, the output image in Fourier spectrum can be obtained by multiplying input signals with repeated

Fourier transforms of E_H, as $\mathcal{F}\left\langle \mathcal{F}\left\{E_H\left(x',y'\right)\right\}\right\rangle = E_H\left(-x',-y'\right)$. Hence, as the ratio of the output signal and input signal in Fourier spectrum, E_H acts as transfer function and x' and y' indicate the spatial Fourier frequencies (wavevectors). Huygens' metasurface can directly modulate the spatial Fourier spectrum through E_H for predesigned analog processing. For direct expression of formula in the main text, Equation (3) can be described as:

$$E_1\left(x_1,y_1\right)=\left\{\frac{k^2}{2\pi f_1 f_2}\exp\left[ik\left(f_1+f_2\right)\right]\exp\left[\frac{ik}{2f_2}\left(x_1^2+y_1^2\right)\right]\right\}\left\{E_0\left(-\frac{f_1}{f_2}x_1,-\frac{f_1}{f_2}y_1\right)\phi\left(x_1,y_1\right)\right\}$$
$$\odot\mathcal{F}\left\{E_H\left(x',y'\right)\right\}\left[k_x,k_y\right] \quad (4)$$

where the additional phase factor $\phi\left(x_1,y_1\right)=\exp\left[\frac{ikf_1}{2f_2^2}\left(x_1^2+y_1^2\right)\right]$ and $k_x=\frac{k}{f_2}x_1$ and $k_y=\frac{k}{f_2}y_1$. Overall, by superimposing the specific phase factor related with the input and output focal length $\exp\left[-\frac{ik}{2f}\left(x'^2+y'^2\right)\right]$ on the transfer function E_H algorithmically, the proposed Huygens' metasurface can directly manipulate spatial Fourier frequencies for the target outputs with single-layer structure.

Supplementary Note 3 Design of Huygens' meta-atom structure

Huygens' metasurface is a two-dimensional array composed of crossed magnetic and electric dipoles which can tailor the scattered electric and magnetic field distribution. Based on Huygens' principle and boundary conditions, the relationship between the surface impedance of electric, magnetic dipoles and the value of reflection, transmission coefficient is derived as[1, 2]:

$$Z_e = \frac{\eta}{2}\frac{1+(R+T)}{1-(R+T)} \tag{5}$$

$$Z_m = 2\eta\frac{1+(R-T)}{1-(R-T)} \tag{6}$$

where $R = re^{j\phi_r}$ and $T = te^{j\phi_t}$ are the predetermined refection and transmission coefficient. Z_e and Z_m denote the surface electric and magnetic impedance respectively. For the design of transsmission-type Huygens' metasurface, Equation (5) and (6) are expressed as

$$Z_e = \frac{\eta}{2}\frac{1+te^{j\phi_t}}{1-te^{j\phi_t}} \tag{7}$$

$$Z_m = 2\eta\frac{1-te^{j\phi_t}}{1+te^{j\phi_t}} \tag{8}$$

where the reflection coefficient remains 0 for the minimization of reflected energy. ϕ_t represents transmission phase ranging from 0 to 2π, while t denotes transmission amplitude ranging from 0 to 1. η is set to be 377Ω as the impedance of the free space. Hence, Equation (7) and (8) indicate that the independent and complete control of transmission amplitude and wavefront can be realized by modulating the surface impedance of resonant electric and magnetic dipole, which are constructed by predesigned metal patches in microwave regime. Particularly, for the phase-only manipulation, Huygens' metasurface can enable the full-phase manipulation with 100% transmission efficiency in theory.

The proposed Huygens' meta-atoms working at 10 GHz are simulated using the commercial software CST Microwave Studio and the results are depicted in Supplementary Fig. 1. The split-ring resonator operates as the magnetic dipole with an induced surface current flowing in a loop. On the other side of the substrate, the electric-LC resonator plays the role of the electric dipole with the main surface current flowing along the incident polarized electric field direction, since the capacitive currents are approximately equivalent and flow in opposite direction with respect to the inductor. The magnetic field introduced by the current loops are offset. Therefore, by appropriately changing the length of the resonators, surface electric and magnetic impedance can be adjusted to achieve the desired transmission coefficient.

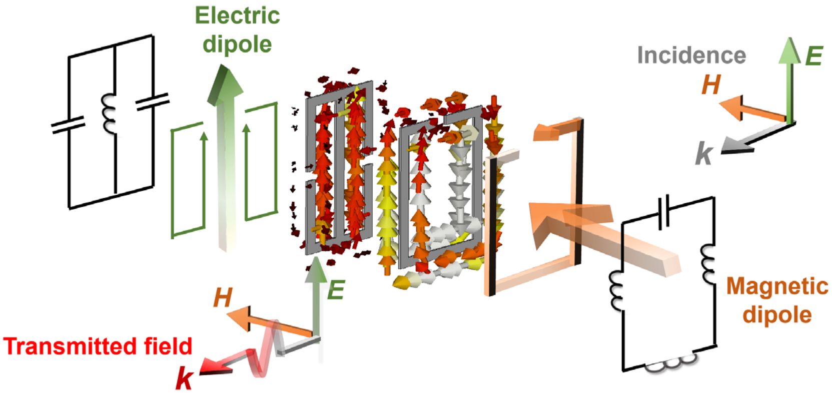

Supplementary Figure 1. Analysis of Huygens' metaatom structure. The simulated surface current distribution and the corresponding effective electric circuits on the metallic patches.

Supplementary Table 2 illustrates 25 sets of geometrical parameters (including l_e, h_e and l_m, h_m) in the proposed Huygens' meta-atom structures, which manipulate the inductance values of electric-LC resonator and magnetic split-ring resonators for the discretization of full phase shift and amplitude control.

Supplementary Table 2. 25 Huygens' meta-atoms with different geometrical parameters (l_e, h_e and l_m, h_m)

	#1	#2	#3	#4	#5	#6	#7	#8	#9
l_e (mm)	1.8	2.2	1	2.6	2	1.2	3	2.8	2.4
h_e (mm)	3.3	3.3	2.1	3.3	3.3	3.3	3.3	3.3	3.3
l_m (mm)	4.2	4.4	3.4	3.2	3.2	0.8	5.4	2.4	0.6
h_m (mm)	3	3	3	3	2.4	3	3	3	3
	**#10**	**#11**	**#12**	**#13**	**#14**	**#15**	**#16**	**#17**	**#18**
l_e (mm)	2	1	0.2	1.6	5.8	3.4	3.8	2.8	2.4
h_e (mm)	3.3	3.3	2.5	3.3	3.3	3.3	3.3	3.3	3.3
l_m (mm)	3.6	3.2	3.2	3.6	2	5.8	2	3	3
h_m (mm)	3	3	3	2.6	3	3	2.4	3	3
	**#19**	**#20**	**#21**	**#22**	**#23**	**#24**	**#25**		
l_e (mm)	2.4	2	1	0.6	0.4	0.6	3.8		

h_e (mm)	3.3	3.3	3.3	3.3	3.3	3.3	3.3		
l_m (mm)	3	3	3	2.6	0.6	0.6	3.8		
h_m (mm)	3	3	3	3	3	3	3		

Supplementary Note 4 The wavefront profiles on Huygens' metasurface for edge detection

Supplementary Fig. 2 demonstrates the transmission amplitude and transmission phase distributions on Huygens' metasurfaces corresponding to four designated image processing techniques associated with the extraction of edge information, including x-axis edge detection, y-axis edge detection, vertex detection and 2D edge detection. According to Equation (2), the wavefront profiles can be obtained by superimposing the phase factor $\exp\left[-\frac{ik}{2f}\left(x'^2+y'^2\right)\right]$ and the transfer function derived in the main manuscript based on derivative property of Fourier transform, as

$$E_{meta}\left(x',y'\right)\propto\exp\left[-\frac{ik}{2f}\left(x'^2+y'^2\right)\right]\times\left(jx'\right)$$ for x-axis edge detection,

$$E_{meta}\left(x',y'\right)\propto\exp\left[-\frac{ik}{2f}\left(x'^2+y'^2\right)\right]\times\left(jy'\right)$$ for y-axis edge detection,

$$E_{meta}\left(x',y'\right)\propto-\exp\left[-\frac{ik}{2f}\left(x'^2+y'^2\right)\right]\times\left(x'y'\right)$$ for vertex detection,

and $E_{meta}\left(x',y'\right)\propto-\exp\left[-\frac{ik}{2f}\left(x'^2+y'^2\right)\right]\times\left[j\left(x'+jy'\right)\right]$ for 2D edge detection.

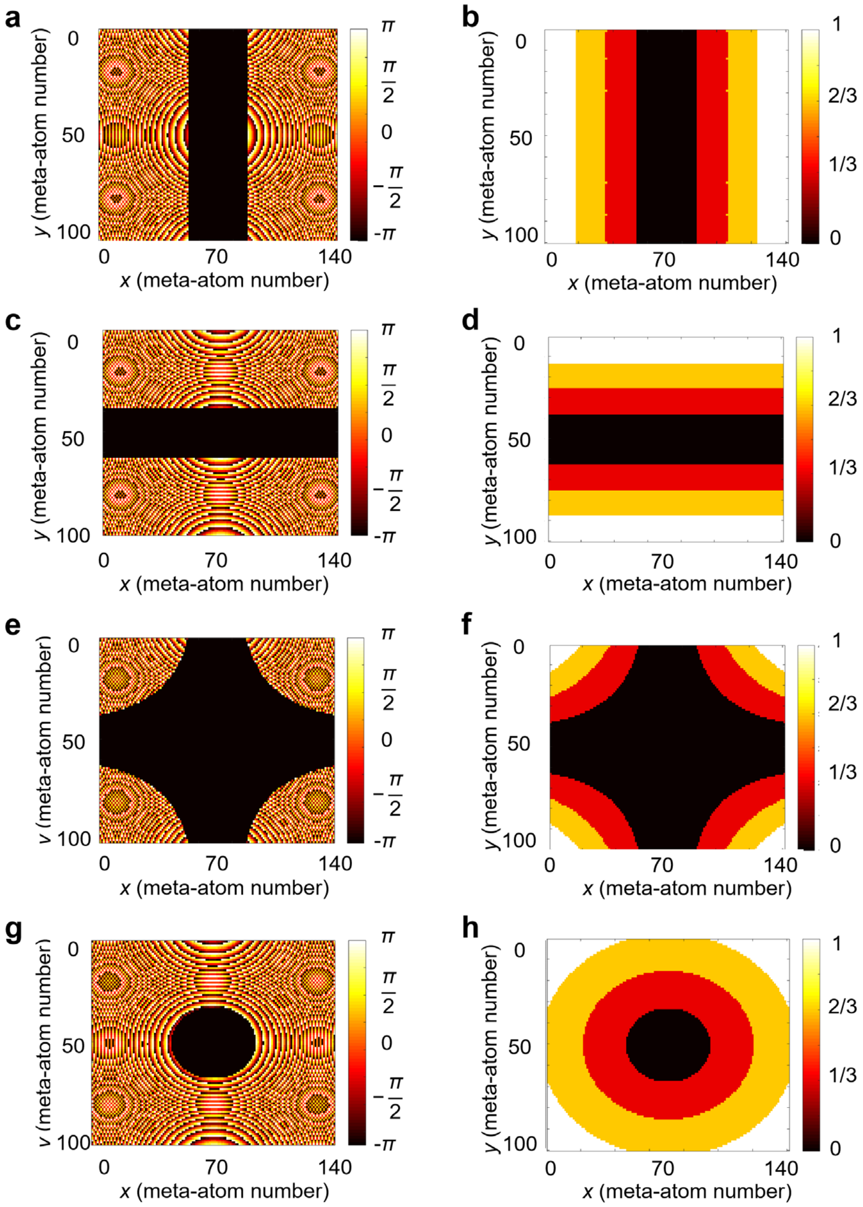

Supplementary Figure 2. Derivative operation. (a) The phase profile and (b) The amplitude distribution on the 100 × 140 Huygens' metasurface for the first-order derivative operation along x-axis. (c) The phase profile and (d) The amplitude distribution on the 100 × 140 Huygens' metasurface for the first-order derivative operation along y-axis. (e) The phase profile and (f) The amplitude distribution on the 100 × 140 Huygens' metasurface for 2D edge detection. (g) The phase profile and (h) The amplitude distribution on the100 × 140 Huygens' metasurface for vertex detection.

Supplementary Note 5 The resolution of the first-order derivative and cross-correlation operation

As an indicator of the proposed analog processor, the system resolution of edge detection and cross-correlation operation is analyzed respectively. As shown in Supplementary Fig. 3a and 3b, the 2D edges of rectangles with different sizes are clearly demonstrated along both horizontal and vertical directions When the minimal edge length decreases to 20mm, the detected edge revels intermittent and a dramatic increase of sidelobe intensity influences the edge extraction. Hence, the resolution of the proposed first-order derivative operator resolution can be considered to be roughly 25mm.

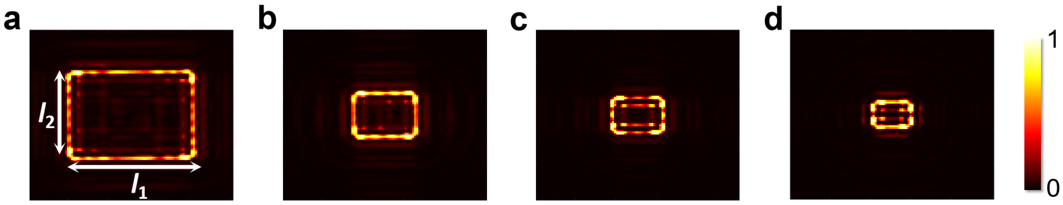

Supplementary Figure 3. The resolution of first-order derivative operation. Output electric field intensity of 2D-edge detection on rectangles with different sizes of (a) l_1=36*mm*, l_2=30*mm*, (b) l_1=30*mm*, l_2=25*mm*, (c) l_1=25*mm*, l_2=20*mm* and (d) l_1=25*mm*, l_2=20mm.

Moreover, the cross-correlation operation between the input with two squares and reference image with one rectangular pattern is analyzed. As shown in Supplementary Fig. 4, the two rectangular pulse of width can not be distinguished until the interval w_2 reaches to the resolution of 40*mm*."

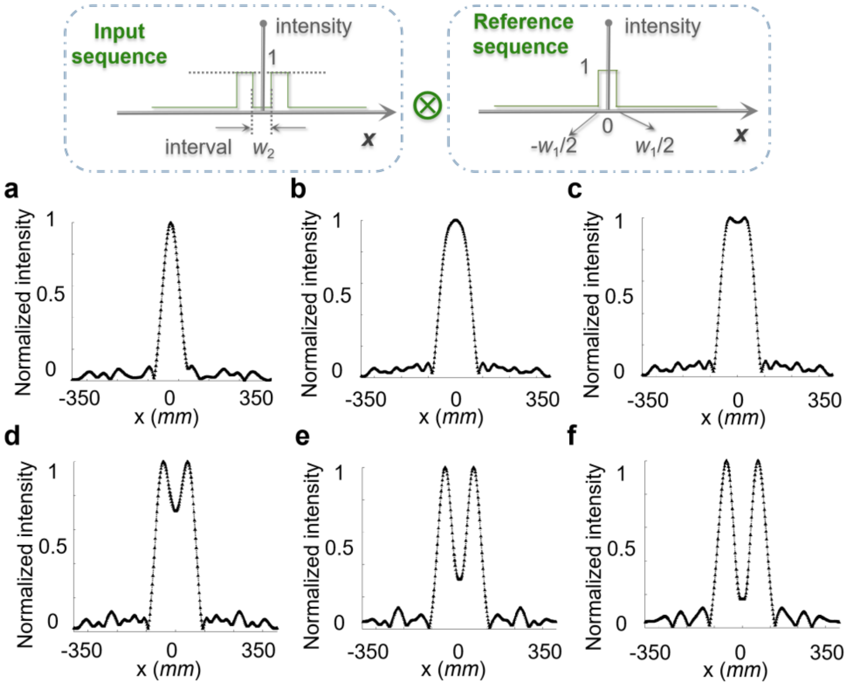

Supplementary Figure 4. The resolution of cross-correlation operation. Output electric field intensity of the cross-correlation between the one-rectangular-pattern sequence and the input two-

square sequence with width w_1= 70*mm* and interval (a) w_2=20*mm*, (b) w_2=38*mm*, (c) w_2=40*mm*, (d) w_2=50*mm*, (e) w_2=70*mm*, (f)w_2= 90*mm*.

Supplementary Note 6 Influence of phase and amplitude discretization on the performance of 2D-edge detection

The influence of phase and amplitude discretization on the Huygens' metasuface-based processor is analyzed from the examples of 2D-edge detection. As shown in Supplementary Fig. 5, the processor with nearly smooth phase and amplitude modulations gives the sharpest feature of the edge. With the decrease of quantization level of complex-wavefront modulation, the edges are still apparent, but show wider peaks, more significant side lobes and more background noises, especially under two-level phase modulation and four-level amplitude modulation (2PM-4AM) and 8PM-2AM. The results validate the fact that the output performance can be improved by increasing the phase and amplitude quantization level. "

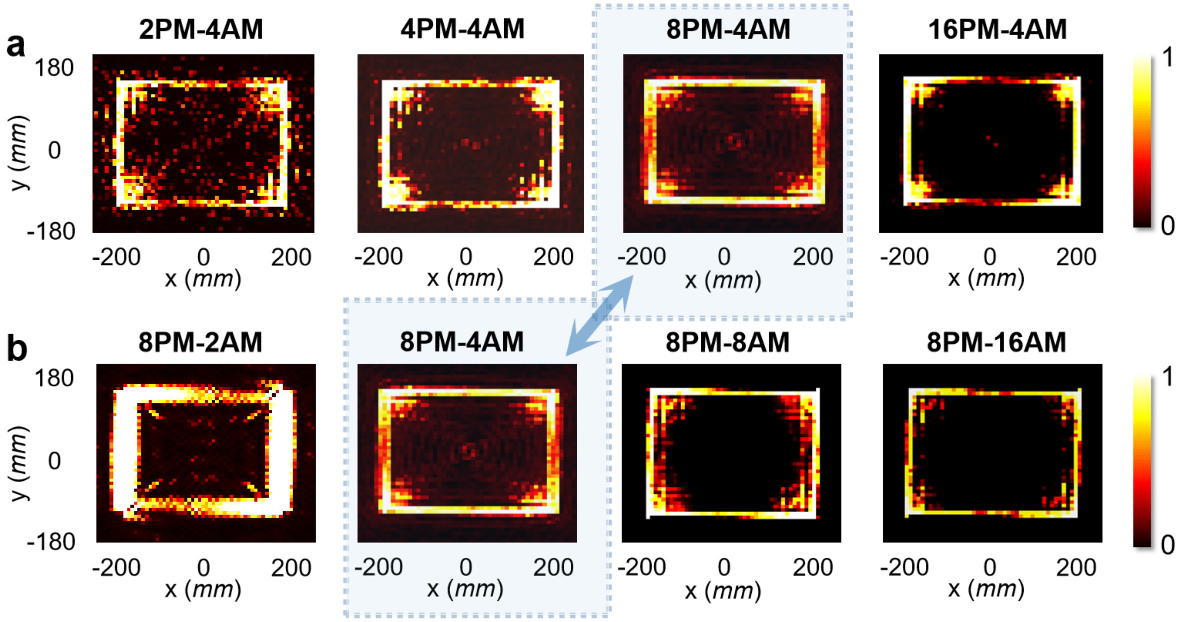

Supplementary Figure 5. Influence of phase and amplitude discretization on the performance of 2D-edge detection. (a) The output electric field intensity distribution utilizing Huygens' metasurface with different phase modulation and amplitude modulation levels: 2PM-4AM, 4PM-4AM, 8PM-4AM, 16PM-4AM respectively for the analysis of phase quantization. (b) The output electric field intensity distribution utilizing Huygens' metasurface with 8PM-2AM, 8PM-4AM, 8PM-8AM, 8PM-16AM respectively for the analysis of amplitude quantization. The cases highlighted in pale blue color in the (a) and (b) are the same 8PM-4AM quantization configuration selected in the main text.

Supplementary Note 7 The wavefront profiles on Huygens' metasurfaces for cross-correlation on one-dimensional sequence

For the validation of the proposed cross-operator, three reference sequences ①, ② and ③ are built by Huygens' metasurfaces respectively to test the similarity with the input two-square-pulse image. With the same method as the first cross-correlation experiment, the features of three reference sequences are built by designing the transfer function first, as

$$E_H(x', y') \propto \left[L_x \frac{k}{f_2} \text{sinc}\left(L_x \frac{k}{f_2} x'\right)\right] \text{ for ①}$$

$$E_H(x', y') \propto \left[L_x \frac{k}{f_2} \text{sinc}\left(L_x \frac{k}{f_2} x'\right)\right] \times \exp\left(\frac{ik}{f_2} s_1 x'\right) \text{ for ②}$$

$$E_H(x', y') \propto \left[L_x \frac{k}{f_2} \text{sinc}\left(L_x \frac{k}{f_2} x'\right)\right] \times \left[\exp\left(\frac{ik}{f_2} s_1 x'\right) + \exp\left(\frac{ik}{f_2} s_2 x'\right)\right] \text{ for ③}$$

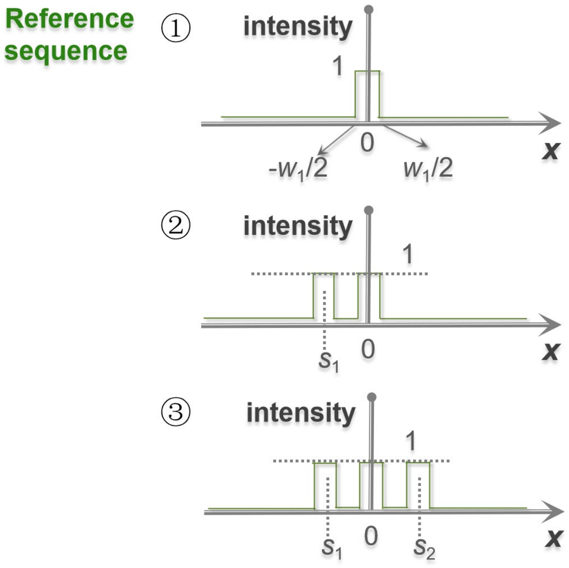

Supplementary Figure 6. **Schematic of one-dimensional sequence for cross-correlation.** Three reference sequences ①, ② and ③ contain one, two or three rectangular pulses respectively.

Then, the complex conjugate operation of $E_H(x)$ is performed to transfer the convolution equation to cross-correlation relationship for sequence alignment. As shown in Supplementary Fig. 7, according to Equation (2), the aperture function on Huygens' metasurface can be obtained by multiplying the complex conjugate transfer function $E_H^*(x', y')$ with the phase factor $\exp\left[-\frac{ik}{2f}\left(x'^2 + y'^2\right)\right]$, as

$$E_{meta}(x', y') \propto \exp\left[-\frac{ik}{2f}\left(x'^2 + y'^2\right)\right] \times E_H^*(x', y') \text{ for ①, ② and ③.}$$

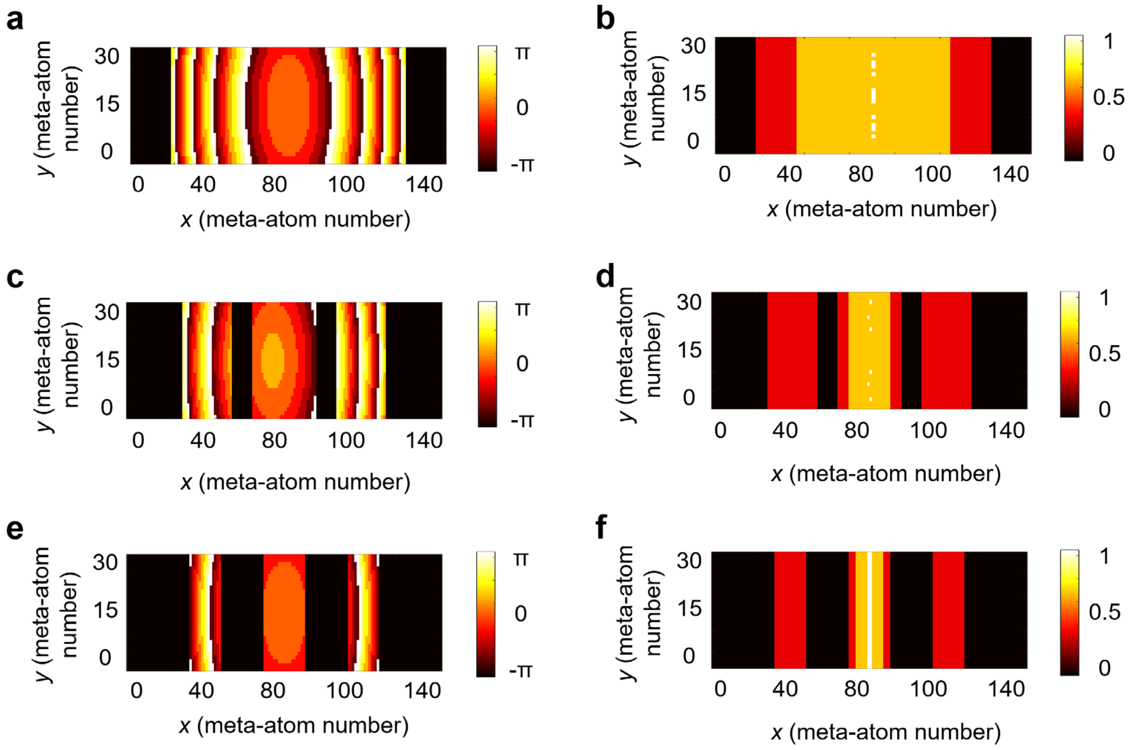

Supplementary Figure 7. Cross-correlation operation. (a) The phase profile and (b) The amplitude distribution on the 140 × 30 Huygens' metasurface for the cross-correlation operation operation on the input two-square sequence and ① sequence. (c) The phase profile and (d) The amplitude distribution on the 140 × 30 Huygens' metasurface for the cross-correlation operation operation on the input two-square sequence and ② sequence. (e) The phase profile and (f) The amplitude distribution on the 140 × 30 Huygens' metasurface for the cross-correlation operation operation on the input two-square sequence and ③ sequence.

Supplementary Note 8 Application of Huygens' metasurface processor at optical frequencies

To enhance the practicability and maneuverability of the Huygens metasurface processor, the proposed working mechanism has been applied to the optical-spectrum edge detection to improve the image quality, and the Laplace operation is implemented to increase function diversity, respectively. Firstly, compared with microwave differentiator, the first-order derivative operation performed at optical frequencies can extract micrometer-scale edges and enable detection of sophisticated images. To detect the details of input image 'H' at λ= 532 *nm*, the input and output focal length are set to be $f_1 = f_2 = 80\lambda$ and the number of meta-atoms is set to be 350 × 350 with periods of 300 *nm*. As shown in Supplementary Fig. 8 a-d, the processed image reveals all edge details and low noise at working wavelength.

Then, the Laplace operator, as the isotropic second-order differentiation ($\nabla^2 E_0(x_1, y_1) = \left| \frac{d^2 E_0(x_1, y_1)}{dx_1^2} + \frac{d^2 E_0(x_1, y_1)}{dy_1^2} \right|$, where ∇^2 indicates the Laplace operator and $E_0(x_1, y_1)$ denotes the input electric field), can be performed by our proposed metaprocessor. According to Equation (2) and (4), by designing the transfer function $E_H(x', y')$ as

$$E_H(x', y') \propto -x'^2 - y'^2, \tag{9}$$

Huygens metasurface can extract the edge details of the 2D object. As shown in Supplementary Fig. 8 e-h, the output numerical results demonstrate the prominent edge features, which indicate the feasibility and flexibility of the proposed Huygens' metasurface processor in various scenarios.

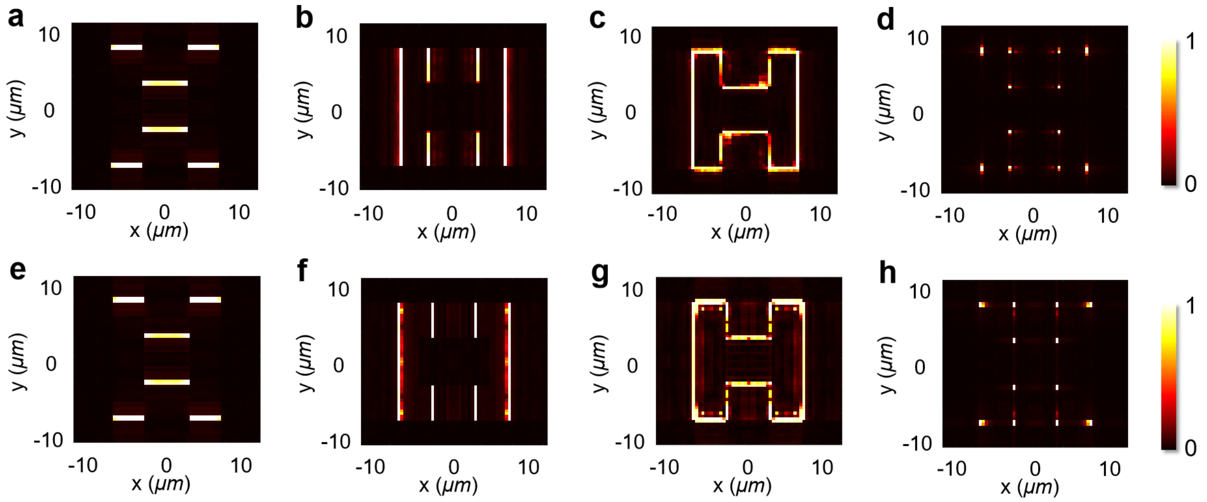

Supplementary Figure 8. Huygens' metasurface processor at optical frequencies. Numerical calculations of normalized electric field intensity distribution processed by the first-order derivative operation for (a) x-axis (b) y-axis (c) 2D edge and (d) vertex detection at λ= 532 *nm*. Numerical calculations of normalized electric field intensity distribution processed by the Laplace operation for (e) x-axis (f) y-axis (g) 2D edge and (h) vertex detection at λ= 532 *nm*.

Supplementary Note 9 Photograph of the fabricated Huygens' metasurfaces

Supplementary Fig. 9 shows the fabricated Huygens' metasurface for edge detection and sequence alignment respectively. The metasurfaces are composed of parallel dielectric on which are printed the Huygens' metaatoms.

a b

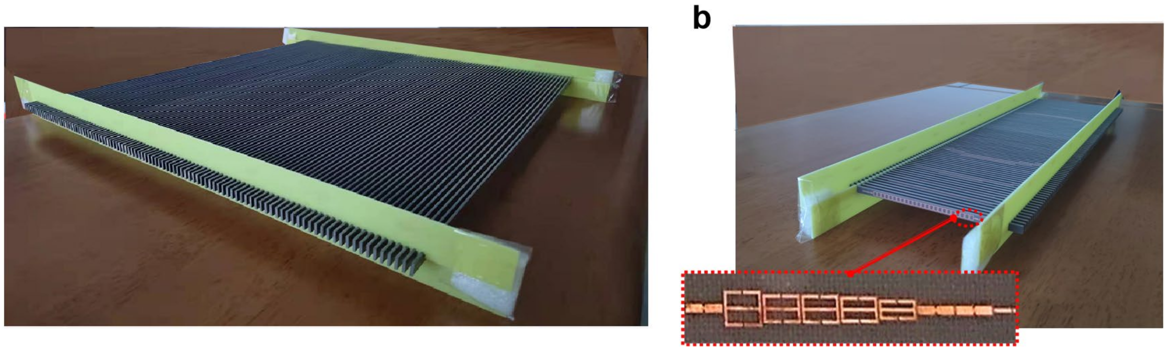

Supplementary Figure 9. The fabricated Huygens' metasurface. (a) The 100×140 Huygens' metasurface for 2D-edge on rectangle (b) The 140×30 Huygens' metasurface for cross-correlation operation.The inset is a zoomed-in view of one-side fabricated Huygens metasurface.

Supplementary References

1. Pfeiffer, C. & Grbic, A. Metamaterial huygens' surfaces: tailoring wave fronts with reflectionless sheets. Phys. Rev. Lett. 110, 197401 (2013).
2. Zhu, B. O. & Feng, Y. Passive metasurface for reflectionless and arbitary control of electromagnetic wave transmission. IEEE Trans. Antennas Propag. 63, 5500-5511 (2015).
